# Expression and functional activity of nucleoside transporters in human choroid plexus

**DOI:** 10.1186/1743-8454-7-2

**Published:** 2010-01-11

**Authors:** Zoran B Redzic, Slava A Malatiali, Danica Grujicic, Aleksandra J Isakovic

**Affiliations:** 1 Department of Physiology, Faculty of Medicine, Kuwait University, P O Box 24923, Kuwait; 2 Institute of Neurosurgery, Clinical Centre of Serbia, Belgrade, Serbia; 3 Department of Biochemistry, School of Medicine, Belgrade, Serbia

## Abstract

**Background:**

Human equilibrative nucleoside transporters (hENTs) 1-3 and human concentrative nucleoside transporters (hCNTs) 1-3 in the human choroid plexus (hCP) play a role in the homeostasis of adenosine and other naturally occurring nucleosides in the brain; in addition, hENT1, hENT2 and hCNT3 mediate membrane transport of nucleoside reverse transcriptase inhibitors that could be used to treat HIV infection, 3'-azido-3'-deoxythymidine, 2'3'-dideoxycytidine and 2'3'-dideoxyinosine. This study aimed to explore the expression levels and functional activities of hENTs 1-3 and hCNTs 1-3 in human choroid plexus.

**Methods:**

Freshly-isolated pieces of lateral ventricle hCP, removed for various clinical reasons during neurosurgery, were obtained under Local Ethics Committee approval. Quantification of mRNAs that encoded hENTs and hCNTs was performed by the hydrolysis probes-based reverse transcription real time-polymerase chain reaction (RT-qPCR); for each gene of interest and for 18 S ribosomal RNA, which was an endogenous control, the efficiency of PCR reaction (E) and the quantification cycle (Cq) were calculated. The uptake of [^3^H]inosine by the choroid plexus pieces was investigated to explore the functional activity of hENTs and hCNTs in the hCP.

**Results:**

RT-qPCR revealed that the mRNA encoding the intracellularly located transporter hENT3 was the most abundant, with E^-Cq ^value being only about 40 fold less that the E^-Cq ^value for 18 S ribosomal RNA; mRNAs encoding hENT1, hENT2 and hCNT3 were much less abundant than mRNA for the hENT3, while mRNAs encoding hCNT1 and hCNT2 were of very low abundance and not detectable. Uptake of [^3^H]inosine by the CP samples was linear and consisted of an Na^+^-dependent component, which was probably mediated by hCNT3, and Na^+^-independent component, mediated by hENTs. The latter component was not sensitive to inhibition by S-(4-nitrobenzyl)-6-thioinosine (NBMPR), when used at a concentration of 0.5 μM, a finding that excluded the involvement of hENT1, but it was very substantially inhibited by 10 μM NBMPR, a finding that suggested the involvement of hENT2 in uptake.

**Conclusion:**

Transcripts for hENT1-3 and hCNT3 were detected in human CP; mRNA for hENT3, an intracellularly located nucleoside transporter, was the most abundant. Human CP took up radiolabelled inosine by both concentrative and equilibrative processes. Concentrative uptake was probably mediated by hCNT3; the equilibrative uptake was mediated only by hENT2. The hENT1 transport activity was absent, which could suggest either that this protein was absent in the CP cells or that it was confined to the basolateral side of the CP epithelium.

## Background

Nucleosides play key roles as precursors for nucleotide synthesis by salvage pathways in a number of human tissues. Their cellular uptake and release are dependent on the activity of one or more members of two families of membrane proteins, the human equilibrative nucleoside transporters (hENTs) and the human concentrative nucleoside transporters (hCNTs). Concentrative nucleoside transport processes are found primarily in specialized epithelial tissues [1]; the major concentrative transport processes, *cit, cif *and *cib*, which are, respectively, pyrimidine nucleoside-preferring, purine nucleoside-preferring and of broad specificity, are mediated in humans by the proteins hCNT1, hCNT2 and hCNT3 [1]. Equilibrative nucleoside transport processes appear to be ubiquitous but differ in their sensitivities to inhibition by the nucleoside analogue S-(4-Nitrobenzyl)-6-thioinosine (NBMPR) and their subcellular localizations. These processes are mediated by the proteins hENT1, hENT2 [2] (with hENT1 being 1000-fold more sensitive to NBMPR inhibition than hENT2 [2]) and hENT3, which is predominantly located intracellularly, in lysosomes [3] and mitochondria [4], although human placental cells expressed this protein in the cell membrane [4].

Several homeostatic mechanisms maintain adenosine concentrations in brain interstitial fluid (ISF) within narrow limits and, therefore, influence adenosine neuromodulatory effects in the CNS: cellular uptake into neurones/glia, with trapping by phosphorylation *via *adenosine kinase (AK, ATP: adenosine 5'-phosphotransferase) (EC 2.7.1.20) [5]; efflux transport across the blood brain barrier (BBB) and metabolic degradation into nucleobases by the brain endothelial cells [6] and the slow bulk flow of brain ISF, driven by the newly formed ISF around the capillaries, which exits towards the cerebrospinal fluid (CSF) [7]. Once adenosine molecules reach the CSF by the bulk flow of ISF, they can then either enter the systemic circulation or the lymph by CSF bulk flow or they can be removed from ventricular CSF into blood by efflux transport across the epithelium of the four choroid plexuses (CPs), which form the blood-cerebrospinal fluid barrier (BCSFB) *in vivo*. Efflux transport of adenosine across the BCSFB depends primarily on three factors: the surface area between the CP epithelium and the CSF, which is expanded by the presence of microvilli on the apical side of the epithelium and complex interdigitations between the lateral walls of the epithelial cells [8]; the presence of nucleoside transporters in the CP epithelium; and the concentration gradient for this nucleoside across that epithelial layer.

In addition to their role in homeostasis of adenosine and other naturally occurring nucleosides, hENTs and hCNTs play a role in membrane transport of several synthetic nucleosides, which act as nucleoside reverse transcriptase inhibitors. Human nucleoside transport protein hENT2, expressed in *Xenopus *oocytes, mediates transport of 3'-azido-3'-deoxythymidine (AZT), 2'3'-dideoxycytidine (ddC), and 2'3'-dideoxyinosine (ddI) across the cell membrane [9]. Strazielle *et al*. have shown [10] that basolateral to apical influx transport of AZT across the CP epithelium was significantly higher (0.6 × 10^-3^cm/min) than influx transport of the extracellular space marker sucrose (0.19 × 10^-3^cm/min); however, those authors also determined that the presence of thymidine in the medium did not affect AZT influx transport. ddI transport from blood into guinea pig choroid plexus involves an organic anion transporting polypeptide 2-like transporter, while its transport across the BBB involves nucleoside transporters [11]. hCNT3 expressed in *Xenopus laevis *oocytes could mediate transport of those synthetic nucleosides, but this activity critically depended on the 3'-hydroxyl position in substrates [12]. Thus, when compared to transport of thymidine via hCNT3, transport of AZT by this transporter was moderate [12,13], while the transport of ddC and ddI was low, when compared to transport of cytidine and inosine by this transporter [12]. hENT3 can transport AZT, ddC, and ddI [3].

In this study we used freshly isolated pieces of human CP to explore the expression of hENTs and hCNTs at the transcript level and to explore the *in vitro *uptake of [^3^H]inosine by the CP. Inosine was chosen for the uptake studies because this nucleoside is a good substrate for hENT1 and 2 [14], hENT3 [3] and hCNT3 [15], but the rate of its intracellular trapping by phosphorylation is much lower than for adenosine. This is mainly because inosine kinase (ATP:inosine 5'-phosphotransferase, EC 2.7.1.73) is absent from mammalian tissues, including the whole brain homogenate [16] and inosine has low affinity for adenosine kinase [17], which is the most abundant nucleoside kinase in mammals.

The results in this study indicate that human CP expressed three equilibrative nucleoside transporter family members, hENTs 1-3, but only one type of concentrative nucleoside transporter, hCNT3; however, the uptake of inosine by freshly isolated CP pieces was found to be mediated primarily by just two transporter types, hENT2 and hCNT3.

## Methods

### Tissue and ethical approval

Pieces of human CP used in this study represented spare material from neurosurgery that, for various clinical indications, had to be dissected from lateral ventricle CPs. A total of 20 samples of apparently pathologically-unaltered CP tissue, 21-48 mg wet weight each, were collected in the Department of Neurosurgery, Clinical Centre in Belgrade, Serbia over a period of four years (2004 - 2008); the age of patients was 20-55 years. This procedure conformed to the standards set by the Declaration of Helsinki and was also in accordance with the Human Tissue Act of 2004. The procedure had been approved by the Ethics Committee of the Clinical Centre Belgrade.

### Radioisotopes and solutions

[2,8-^3^H]Inosine (Specific Activity 20 Ci/mmol) was purchased from ICN Pharmaceuticals (Costa Mesa, CA, USA); [(U)-^14^C]sucrose (Specific Activity: 400-700 mCi (14.8-25.9 GBq mmol^-1^) was purchased from Perkin Elmer (Waltham, Massachusetts, USA). These isotopes were used without further purification. The following solutions were used for tissue processing and uptake studies: Hank's Balanced Salt Solution (HBSS) - based buffer containing (mM) NaCl 138, KCl 5.33, NaHCO_3 _4.17, Na_2_HPO_4 _0.3, KH_2_PO_4 _0.44, D-glucose 5.54, HEPES 5; pH 7.4. Artificial CSF (aCSF) containing (mM): NaCl 121, KCl 2.5, NaHCO_3 _18, urea 2, MgCl_2 _0.8, CaCl_2 _1.4, 2'-deoxy-D-glucose 10, 4-amido-5-iodo-7-(β-D-ribofuranosyl)-pyrrolo [2,3-d] pyrimidine, also known as 5-iodotubercidin (5-IT) 1 μM; pH 7.35.

Low Na^+^aCSF, of the same composition as aCSF, except that NaCl and NaHCO_3 _were replaced by choline chloride and choline bicarbonate, respectively.

### Tissue processing

During the neurosurgery hCP samples were placed immediately in ice cold HBSS - based buffer and examined by a 10× magnification glass. Samples which were obviously altered by various pathological processes were excluded from the study. Tissue was either used for the study immediately thereafter or frozen in liquid nitrogen and kept at -80°C until use.

### Quantification of mRNA encoding nucleoside transporters

Nomenclature used in this manuscript was according to the "Minimum Information for Publication of Quantitative Real-Time PCR Experiments" guideline [18]. Total RNA was isolated from tissue using the standard Trizol/chloroform/isopropanol procedure and RNA pellets were dissolved in RNase-free, diethylpyrocarbonate-treated water at 55°C for 10 min; all samples were checked for RNA integrity and RNA concentrations were determined from absorbance at 260 nm. In all cases, 2 μg portions of total RNA were used for first-strand cDNA synthesis. Intron-spanning primers were used in the study that excluded interference from genomic DNA in the results; however, all RNA samples were treated with DNA-ase, 4 units/sample (Invitrogen, Carlsbad, California, USA) prior to the reverse transcription. Reverse transcription was performed with MuMLV reverse transcriptase (RT) (Invitrogen) using random hexamers (Invitrogen), according to the manufacturer's instructions. The samples in which the RT was replaced by water (RT-ve) were considered as negative controls.

Both RT+ve and RT-ve samples were used for qPCR. In some cases, as explained below, the qPCR reactions were also performed using a human (Raji) cDNA as a control. This cDNA was obtained from immortalized lymphoblastoid cells, derived from a Burkitt's lymphoma (ATCC #CCL86) and was supplied by the manufacturer as a 25 ng.μl^-1 ^template. It was used as a positive control, to prove that the reverse transcription was performed correctly and that the absence of hCNT1 and hCNT2 transcripts in hCP was not due to ineffective hydrolysis probes. In either case (cDNA synthesized from the hCP mRNA or control cDNA), the template was diluted to the final concentration of 250 ng.ml^-1^; then 1 μl cDNA template, which corresponds to 0.25 ng cDNA, was used for the qPCR.

qPCR reactions were carried out in the Applied Biosystems 7500 real time model (Applied Biosystems, Foster City, California, USA) using the following gene expression assays, which had intron-spanning primers and hydrolysis probes labelled with 6-carboxyfluorescein (FAM) as a reporter dye and 6-carboxy-tetramethyl-rhodamine (TAMRA) as a quencher dye: hs01085706 for hENT1, hs00155426 for hENT2, hs00983219 for hENT3, hs00984403 for hCNT1, hs00188407 for hCNT2, hs00223220 for hCNT3 and hs99999901 for h18 S. The reaction mixture was prepared according to the instructions from the manufacturer and consisted of 1 μl cDNA template (corresponded to 0.25 ng cDNA), 1.25 μl primer mix, RNase-free water and Taqman universal master mix (Applied Biosystems) up to 25 μl. The PCR reactions were carried out in a 96-well plate that was sealed with an adhesive transparent foil, employing the thermal profile that was suggested by the manufacturer: 2 min at 50°C (1 cycle); 10 min at 95°C (1 cycle), then 15 s at 95°C followed by 1 min annealing at 60°C for 40 cycles. In some cases, a 5 μl sample of the PCR product after 40 cycles was analyzed by electrophoresis on a 1.5% agarose gel that contained ethidium bromide.

The gene accession numbers, probe and amplicon sequences as well as the amplicon lengths are shown in the Table 1. All samples were assayed and quantification cycle (also known as threshold cycle) values (Cq) were calculated for each gene of interest and for 18 S ribosomal RNA. For every sample those values were calculated as averages of 2 replicates.

**Table 1:** Primer sequences and the expected size of the PCR products.

Gene of interest	NCBI Accession number	Position in the mRNA	Amplicon length	Amplicon sequence	Probe sequence
hENT1	NM_001078174	358-433	75	ACAGATACAAAGCGTCT GGCTT ATCTTCTTCATGCTG GGTCTGGGAACGCTGCTC CCGTGGAATTTTTTCATGA	CAGCCTCAGGACAGATACAAAGCTG

hENT2	NM_001532	1290-1400	110	CAGACGAGGACAGCCGG CTGCTGCCCCTGCTGGTCTGCCTGCGGTTCCTGTTCGTGCCCCTCTTCATGCTGTGCCACGTGCCCCAGAGGTCCC GGCTGCCCATCCTCTTCC	CTTACTTCCTGTGGCCAGACGAGGA

hENT3	NM_018344	827 - 909	82	CCAGGTACTACATGAGGCCTGTTCTTGCGGCCCATGTGTTTTCTGGTGAAGAGGAGCTTCCCCAGGACTCCCTCAGTGCCCC	GGAGTATGCCAGGTACTACATGAGG

hCNT1	NM_201651	391 - 463	72	GAACCTGCAGCCAGCCCTGA GAGCCAGAAGCTTCTG CAGGGAGCACATGCAGCTGTTTCGATGGATCGGCAC	TCCAGATGGAGGAACCTGCAGCCAG

hCNT2	NM_004212	236 - 301	65	TCGGTGGCCTTTCAGCAAAGCAAGAAGTTTCTGCAAAACACACGCCAGATTGTTCAAGAAGATCC	ACTTACCAGAGGAGGAGTCGGTGGC

hCNT3	NM_022127	617-718	101	GGTG ATCTGGAGCTCCCT GGTCCT AGCAGTTATTTTCTG GTTGGCCTTTGACACTGCCAAATTGGGTCAACAGCAGCTGGTGTCCTTCGGTGGG CTCATAA	CTGGCTGAAGTGGGTGATCTGGAGC

18 S	X03205	609-796	187	G GTGCCAGCAGCCGCG GTAATTCCAGCTCCAATAGCGTATA TTAAAGTTGCTGCA GTTAAAAAGCTCGTAGTTGGATCTTGGGAGCGGGCGGGCGGTCCGCCGCGAGGCGA GCCACCGCCCGTCC CCGCCC CTTGCCTCTCGG CGCCCCCTCGATGCTCTTAGCTGAGTGTCCCGCGGGGC CCGAAG	ACTTGCTCTTGGACAGGAACCAGGG

There seem to be two splice variants for hCNT3 [19,20], two splice variants of hENT2 (ENSEMBL entry http://www.ensembl.org/Homo_sapiens/Gene/Summary?db=core;g=ENSG00000174669;r=11:65886569-65895867;t=ENST00000311161), nine splice variants of hENT1 http://www.ensembl.org/Homo_sapiens/Gene/Splice?db=core;g=ENSG00000112759;r=6:44295220-44309866 and only one for hENT3 http://www.ensembl.org/Homo_sapiens/Gene/Summary?g=ENSG00000198246. However, there are no known splice variants or single-nucleotide polymorphism positions documented in transcript and single-nucleotide polymorphism databases in the regions of the genes corresponding to the portions of the cDNA to be amplified in this study according to the ENSEMBL entries or references mentioned above; thus, use of intron-spanning primers could not affect the quantification data in this study.

In some experiments, a series of dilutions of cDNA was made (2-64 fold) and then qPCR reactions carried out as explained above. The obtained Cq values for each dilution for every nucleoside transporter and for 18 S RNA were then plotted against the log dilution. The Pearson product correlation coefficient (r) and slope of the line shaped by those points were calculated and the reaction efficiency (E) was estimated as [21]:(1)

In some experiments PCR products were collected after 40 cycles and were subjected to electrophoresis alongside a DNA ladder (Gibco, Carlsbad, CA, USA), through a 2% (w/v) agarose gel (Boehinger Manheim, Bracknell, Berkshire, UK) and stained with ethidium bromide.

### Uptake of [^3^H]inosine

Fresh tissue was used for the uptake studies; after the dissection into ~2 × 2 × 2 mm pieces, CP tissue was pre-incubated for 30 min in 1 ml of warm (37°C) aCSF or low Na^+^aCSF, gassed with 5% CO_2 _in O_2 _and containing 2-deoxy-D-glucose and 5-IT. CP pieces were then transferred to 0.4 ml aCSF or low Na^+ ^aCSF containing 911 ± 5 disintegrations per minute (DPM) μl^-1 ^(mean ± SEM from 25 samples) of [2,8-^3^H]inosine and 655 ± 3 DPM μl^-1 ^(mean ± SEM from 17 samples) of [(U)-^14^C]sucrose as an extracellular space marker; unlabelled inosine was added to give a final concentration of 2 μM. Where indicated, the nucleoside transport inhibitor NBMPR was added to aCSF at concentrations of either 0.5 μM or 10 μM.

After 1, 5 or 10 min of incubation at 37°C (as indicated in the Results section), the tissue was rinsed in ice-cold aCSF, weighed and solubilised in the tissue solubilizer. The radioactivity in all samples was determined by liquid scintillation counting (LKB Wallac 1219, Turku, Finland) and counts per min were converted into DPM using the internally stored quench curve. The results were first calculated as [^3^H] DPM/mg protein, with the amount of protein in the tissue having been determined according to the Bradford assay [22]. To correct for [^3^H]-radioactivity that remained in the extracellular space of the CP, the [^14^C]DPM/mg protein value, which was multiplied by the [^14^C] DPM/[^3^H] DPM ratio in the sample (a correction factor), was subtracted from the total [^3^H] DPM. Finally, the net [^3^H]radioactivity in the samples was converted to moles inosine mg.protein^-1^, taking into account specific activities of the tracer.

### Statistical analysis

Uptake data are presented as means ± SEM. Analysis of variance (ANOVA) was used to determine the significance of differences between two or several groups. A probability of *p *< 0.05 was taken as the limit of significance.

## Results and Discussion

### Quantification of mRNA for nucleoside transporters

**Figure 1:**
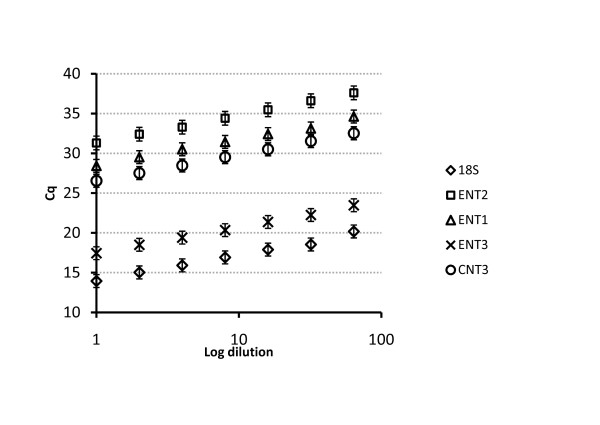
**Validation of the method for fluorescence-based quantitative real-time PCR (qPCR)**. Titration curves were obtained by using cDNA to create a series of dilutions, from 1× (no dilution) to 64 × dilution. Then the qPCR was run and log dilutions plotted against the obtained quantification cycle (Cq) values. Each Cq value was estimated as an average from 2 replicates and each point in the Figure represents mean ± SE from 3 separate cDNA samples. The linearity of the plots shows the equal amplification of the assay over a range of input DNA concentrations. These data were also used to estimate the efficiency (E) of the reaction for each gene of interest.

In order to show that the qPCR assays were quantitative within the range of the cDNA input, as well as in order to estimate the efficiencies of the qPCR reactions, a series of dilutions of CP cDNA was prepared and then the quantification cycle, Cq, values determined for 18 S RNA and for each gene of interest; a plot of log dilutions against the obtained Cq values (Figure 1) demonstrated a linear increase of Cq values with dilution with the linear correlation coefficients 0.9949, 0.9905, 0.9933, 0.9927 and 0.9932 for 18 S RNA, hENT1, hENT2, hENT3 and hCNT3, respectively. The amplification efficiencies (E) under those conditions were 2.01, 1.93, 2.01, 2.02 and 2.00 for 18 S RNA, hENT1, hENT2, hENT3 and hCNT3, respectively.

Table 2 shows the average Cq values of nucleoside transporters in hCP as well as the linear form of the Cq values, E^-Cq^. A total of 5 cDNA samples (obtained from 5 hCP samples) were measured. The Cq values in RT-ve samples were above 40 (not shown in Table 2). The Cq values in RT+ve samples for hCNT2 were above 40, so they were not calculated either. The Cq values for hCNT1 in 3 samples were above 40, while in 2 remaining samples they were 38.6 and 39.4, indicating the absence and very low abundance of mRNA, respectively. The Cq values in RT+ve samples for hENTs 1-3 (29.0 ± 0.52, 28.08 ± 0.30, 19.17 ± 0.59) and for hCNT3 (27.67 ± 0.43, Table 2) were much below the Cq values in RT-ve samples. The Cq values in the control human (Raji) cDNA for 18 S ribosomal RNA, hENT1, hENT2, hENT3, hCNT1, hCNT2 and hCNT3 were (mean ± SD, from 3 repeats) 14.6 ± 0.5, 27.1 ± 0.4, 25.2 ± 0.5, 25.6 ± 0.3, 38.8 ± 0.9, 39.0 ± 0.4 and > 40, respectively.

**Table 2:** The fluorescence-based quantitative real-time PCR data for the reference gene and genes of interest in human choroid plexus.

	Cq ± SD (n = 5)	E^-Cq ^± SD (× 10^9^)
18 S	13.97 ± 0.31	58123 ± 12920
hENT1	29.00 ± 0.52	5.23 ± 1.79
hENT2	28.08 ± 0.30	3.07 ± 0.62
hENT3	19.17 ± 0.59	1403 ± 494
hCNT1	> 40	-
hCNT2	> 40	-
hCNT3	27.67 ± 0.43	4.69 ± 1.35

Values are presented as mean ± SD from 5 separate CP samples. For every sample the quantification or threshold cycle (Cq) value was calculated as an average from 2 replicates. The linear form of the Cq values was corrected for the reaction efficiency (E), and is presented as E^-Cq^. The Cq value was much lower for hENT3 than for other ENTs and CNTs, indicating that this nucleoside transporter was the most abundant at the transcript level.

**Figure 2:**
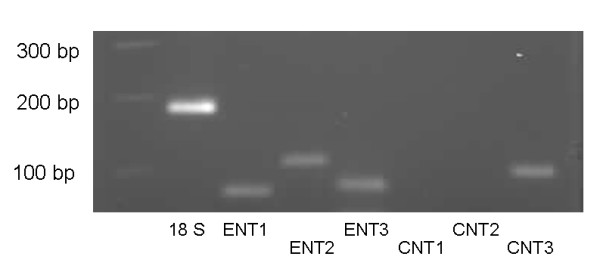
**A typical agarose gel obtained after electrophoresis of the PCR products, stained with ethidium-bromide**. Bands of expected sizes were obtained for hENT1, hENT2, hENT3 and hCNT3, while bands corresponding to hCNT1 and hCNT2 were absent.

Electrophoresis of the PCR products revealed bands of expected sizes for hENT1, hENT2, hENT3 and hCNT3 (Figure 2), while bands corresponding to hCNT1 and hCNT2 were absent.

The obtained E^-Cq ^values (Table 2) indicated that by far the most abundant mRNA was that for hENT3, with the amount of mRNA being only about 40 fold less than the amount of 18 S RNA copies; the hENT1, hENT2 and hCNT3 mRNAs were at least 10^2 ^fold less abundant than the amount of hENT3 mRNA. It has been reported that the hENT3 protein was found to co-localize with lysosomal markers and showed broad selectivity and low affinity towards the naturally occurring nucleosides [3]; the later report indicated that hENT3 was in several human cell lines also a mitochondrial transporter [4], which can also transport several nucleoside reverse transcriptase inhibitors, including AZT, ddI and ddC [4]. Immunoreactivity at cellular membrane was also detected, but only in human placental cell lines [4]. This transporter is relatively insensitive to NBMPR and is strongly dependent upon pH, with its maximum activity at pH 5.5 [3,4]. Bearing in mind that the mitochondrial matrix pH in mammalian cells is slightly alkaline and that the local pH in the intracristal compartments is acidic [23], a condition required for optimal function of the ATP synthase [24], such a gradient provides good conditions for hENT3 activity. A physiological role for hENT3 may be in salvaging nucleosides and deoxynucleosides into the mitochondria, which may be followed by subsequent phosphorylation by intramitochondrial kinases. This process may play a role in mitochondrial DNA synthesis and repair in CP epithelium. This role was also suggested in a report describing two missense mutations and one single nucleotide deletion in the hENT3 gene as a cause of an autosomal recessive disorder called the H syndrome [25].

## Uptake of ^3^H-inosine

**Figure 3:**
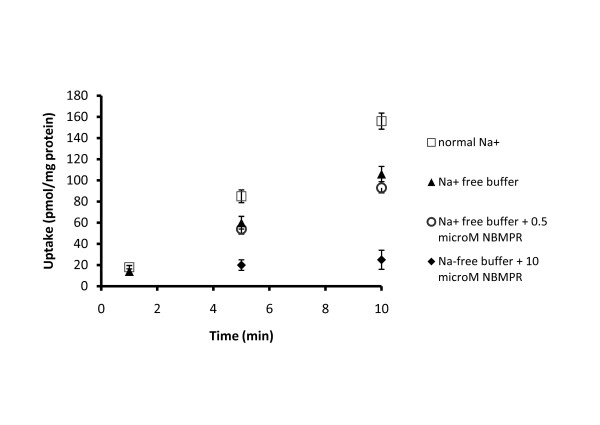
**The uptake of [^3^H] inosine into human CP incubated in aCSF containing 139 mEq Na^+ ^(squares), Na^+^-free aCSF medium (triangles), and Na^+^-free aCSF medium containing either 0.5 μM S-(4-Nitrobenzyl)-6-thioinosine (NBMPR) (circle) or 10 μM NBMPR (deltoid)**. The data are presented as means ± SEM from 3-4 separate experiments. The uptake values for inosine by CP tissue in aCSF (presence of Na^+^) after 5 and 10 min of incubation were significantly higher than the corresponding uptake values seen in Na^+^-free aCSF (*p *< 0.01). Uptake of inosine after 5 min (n = 3) and 10 min (n = 3) of incubation in the presence of 0.5 μM NBMPR in Na^+^-free aCSF did not differ significantly from the corresponding uptake after incubation in Na^+^-free aCSF which did not contain NBMPR (*p *> 0.05). The presence of 10 μM NBMPR in Na^+^-free aCSF caused significant inhibition of inosine uptake after 5 min (n = 4, p < 0.01) and after 10 min (n = 3, *p *< 0.001), when compared to the uptake in Na^+^-free aCSF which did not contain NBMPR.

To avoid saturation of the transport systems, nucleoside uptake studies were performed using a concentration of inosine of 2 μM, which was substantially less than the reported *K*_*m *_values of approximately 170 μM and 50 μM for hENT1 and hENT2 respectively [2]. The uptake values for inosine by CP tissue in the aCSF (presence of Na^+^) after 5 and 10 min of incubation were significantly higher than the corresponding uptake values seen in Na^+^-free aCSF (*p *< 0.01, Figure 3). Uptake of inosine after 5 min (n = 3) and 10 min (n = 3) of incubation in the presence of 0.5 μM NBMPR in Na^+^-free aCSF did not differ significantly from the corresponding uptake after incubation in Na^+^-free aCSF which did not contain NBMPR (Figure 3, *p *> 0.05). However, the presence of 10 μM NBMPR in Na^+^-free aCSF caused significant inhibition of inosine uptake after 5 min (n = 4, p < 0.01) and after 10 min (n = 3, *p *< 0.001), when compared to the uptake in Na^+^-free aCSF which did not contain NBMPR.

The hENT1 and 2 proteins as well as hCNTs are primarily located in the plasma membrane. However, these transporters differ in that hENT1 is inhibited by nanomolar concentrations of NBMPR (IC_50 _0.4 ± 0.1 nM), while hENT2 is ~7000-fold more resistant to inhibition (IC_50 _2.8 ± 0.3 μM) [2]. The observation in the present study that 0.5 μM NBMPR failed to cause significant inhibition of [^3^H]inosine accumulation into CP pieces (Figure 3) indicated that the hENT1 protein, which was present at the transcript level, did not contribute to nucleoside uptake by the CP pieces *in vitro*.

There are several possible explanations for this finding: one of those could be that hENT1 is confined to the basolateral membrane of the CP epithelium; our previous study on rat CP epithelium in primary culture has also shown that rENT1 was confined to the interstitial fluid - facing membrane [26]. The rationale behind this explanation is that the uptake study was performed on isolated samples of human CP, thus no perfusion pressure was present in the microcirculation, a situation which could cause CP capillaries to collapse. Bearing in mind the histological structure of the CP, the diffusion of solutes from the aCSF into the CP interstitial fluid under these circumstances would be rather limited. On the other hand, the existence of tight junctions between epithelial cells of the CP occludes paracellular space and further impedes diffusion of solutes from the aCSF into the CP interstitial fluid that surrounds the basolateral membrane. Therefore, exposure of the basolateral side of the CP epithelium, which faces CP interstitial fluid and CP capillaries, to radiotracers in the aCSF could be considered fairly limited in this study.

Uptake of [^3^H]inosine was reduced significantly by the presence of 10 μM NBMPR (Figure 3); this pattern of inhibition is consistent with involvement of hENT2 in the uptake process. This is in accordance with presence of mRNA encoding hENT2. A role for other transporters that are weakly inhibited by NBMPR could be excluded. Inosine is a permeant for hENT3 [3], which was the most abundant nucleoside transporter at the transcript level in the human CP (Table 2). However, it is unlikely that this transporter contributed to the accumulation of [^3^H]inosine in the CP pieces because of its cellular localization and pH dependence.

Functional uptake studies also revealed that depletion of Na^+ ^from the aCSF caused significant reduction (~25-30%) of inosine uptake by human CP (Figure 3), which was consistent with the involvement of hCNTs. The RT-qPCR data revealed that mRNA for hCNT3 was present in CP samples while mRNAs for hCNT1 and for hCNT2 were not detectable. This finding indicates that the Na^+^-dependent uptake was probably mediated by hCNT3, a transporter that is broadly selective and transports both purine and pyrimidine nucleosides into cells, employing a 2:1 Na^+ ^: nucleoside coupling ratio [15].

Inosine is a natural substrate for inosine kinase (IK); it was initially believed that this enzyme was also present in mammalian tissues [27,28], but it was revealed later that inosine phosphorylation was in fact due to the phosphotransferase activity of cytosolic 5'-nucleotidase [29]; thus, IK (and guanosine kinase) were absent in several mammalian tissues, including whole brain homogenate [16]. On the other hand, adenosine kinase (AK) is the most abundant nucleoside kinase in mammalian tissues, with high affinity for its natural substrate, adenosine (Km 1.98 microM - [5]). It was believed that AK does not have any inosine kinase activity [30]. However, it was found later that inosine analogues could be substrates for the AK, but that depended on the ionization of N1 atom, which in turn depended on pH [17]. The substitution of an N-atom for the C-8 atom of this nucleoside lowers the pKa, so that the N-1 proton is more ionized at pH 7.00; this ionized form resembled adenosine with respect to the bond structure at the 1 and 6 positions of the purine ring, whereas the un-ionized form (as it is in inosine itself) does not [17]. Thus, since inosine pKa is 8.74, the rate of its phosphorylation by AK at pH 7.00 is very low because N-1 proton is 2% ionized; however, at higher pH that rate increases. The pH of the artificial CSF in our study was 7.3-7.4, at that pH the N1 of inosine is more ionized, so we believed that this nucleoside could be, to some extent, a substrate for the AK. That was the rationale behind inhibition of AK in our study by 5-IT, which was present at a concentration which was sufficient to completely inhibit AK according to the published half maximal inhibitory concentration (IC_50_) [31].

Mammalian tissues also contain several other kinases that show various affinities for purine ribo- and deoxyribonucleosides [32]; all those enzymes have ATP as a donor of activated phosphate. Thus, prior to the uptake studies, a 30 min pre-incubation was performed in a glucose-free aCSF, which contained 10 mM 2'-deoxy-D-glucose, an analogue which cannot be metabolized beyond the 6-phosphate form and therefore competitively inhibits glycolysis and the resulting production of pyruvate. It has been shown that incubation of astrocytes and neurons with 10 mM 2'-deoxy-D-glucose in the absence of D-glucose (the strategy that was used in this study) caused depletion of > 90% of cellular ATP [33], while ATP content in lymphocytes after 1 h incubation in medium containing both 2'-deoxy-D-glucose and D-glucose was reduced only 20-30% [34]. Several lines of evidence suggest that glucose is the main fuel for the CP epithelium: human CP epithelium abundantly expresses GLUT1 mainly in the basolateral membrane [35]; rat CP and ependyma had the highest expression of GLUT1 in the brain [36].

It should be noted that the plasma membrane monoamine transporter (PMAT) can, at pH 5.5, also transport naturally occurring nucleosides, thus it is alternatively known as hENT4 [37]. However, expression of this transporter was not investigated because this monoamine transporter has a poor ability to transport purine nucleosides at 7.3-7.4. The pH of human CSF is 7.31-7.35 [38] and the pH does not go below 6.8 even in metabolic acidosis [38], acute respiratory acidosis [39], head injury [40] and brain hypoperfusion [41].

## Conclusions

This study described the presence of individual nucleoside transporters at the transcript level and their functional activities in fresh human CP. The most abundant nucleoside transporter at the transcript level in hCP was hENT3; this tissue also expressed hENT1, hENT2 and hCNT3 at the level of transcripts. Both concentrative and equilibrative nucleoside transport processes were clearly detectable, the latter was probably mediated by hENT2, while ENT1-mediated transport could not be detected.

## Competing interests

The authors declare that they have no competing interests.

## Authors' contributions

ZR - has extracted RNA, performed PCR study (together with SM) and uptake study (in some cases together with AI) and wrote a draft of the manuscript. SM - has performed PCR analysis, together with ZR. DG has collected and did the screening of CP samples. AI - has performed uptake studies with ZR. All authors have read and approved the final version of the manuscript.
